# Gone with Water or Mountain: The Population Genetic Diversity of *Rhopalopsole sinensis* Yang and Yang, 1993 in China

**DOI:** 10.3390/insects16030244

**Published:** 2025-02-26

**Authors:** Qing-Bo Huo, Yu-Ben Yang, Anna Eichert, Yu-Zhou Du

**Affiliations:** 1 Institute of Applied Entomology, College of Plant Protection, Yangzhou University, Yangzhou 225009, China; jw30th@163.com (Q.-B.H.); yubenyang1998@163.com (Y.-B.Y.); 2 Richard Gilder Graduate School, Division of Invertebrate Zoology, American Museum of Natural History, 200 Central Park West, New York, NY 10024-5192, USA; aeichert@amnh.org; 3 Joint International Research Laboratory of Agriculture and Agri-Product Safety, The Ministry of Education, Yangzhou University, Yangzhou 225009, China

**Keywords:** plecoptera, leuctridae, *Rhopalopsole*, population genetics, identification, China

## Abstract

*Rhopalopsole sinensis* Yang and Yang, 1993 is one of the most widely distributed stoneflies in southern China. In this study, the genetic structure of nine different geographical populations of *R. sinensis* was studied based on three datasets of the *18S*, *COI* and *ITS2* genes. The phylogenetic trees showed that there had been a great degree of genetic differentiation among its nine geographical populations, but the hypothesis of random mating did not support the formation of reproductive isolation among different geographical populations, indicating that *R. sinensis* did not form cryptic species. The establishment of the *18S* gene and *ITS2* gene supports the population of (Guangdong + Shaanxi + Sichuan + Guizhou + Fujian) as independent branches, while the population of (Anhui + Zhejiang + Hunan) is another branch. The establishment of *COI* gene tree supports (Shaanxi + Sichuan + Guizhou + Fujian) as one branch, while the other independent branch is composed of (Anhui + Zhejiang + Guangxi + Hunan + Guangdong) groups. The possible reasons for this situation based on geography and biology are discussed. Our results provide the first reference for the genetic differentiation in Chinese *Rhopalopsole* stoneflies.

## Simple Summary

It is commonly considered that the distribution of stoneflies is closely related to water systems. Many species of them are geographically restricted, but there are also some widely distributed taxa. This article presents questions occurred in *Rhopalopsole sinensis* from China: If a widely distributed stonefly species shows almost no morphological differences but exhibits significant genetic distance, could there be cryptic species among them? Additionally, if some populations of the species are more closely related to others that are geographically distant, but less related to the populations from neighboring areas, is this reasonable? Our research in molecular analysis, biogeography and ecology will provide an integrative insight to explain and discuss these cases.

## 1. Introduction

With c.a. 4232 described species across 17 families [1,2,3], the ancient hemimetabolous insect order Plecoptera [4], commonly known as stoneflies, represents an important component of freshwater ecological systems in terrestrial and aquatic food webs [5,6]. Due to their poor dispersal capacity [7], stoneflies are ideal organisms for biogeographical studies [8,9,10,11,12]. Stoneflies are considered one of the most sensitive groups to environmental changes in freshwater ecosystems, and anthropogenic change stemming from altered climate regimes have caused range contractions in many species. Given this critical threat to stoneflies, the study of their distribution, morphological variability, and genetic diversity should be one of the priorities in conservation biology [13].

**Figure 1:**
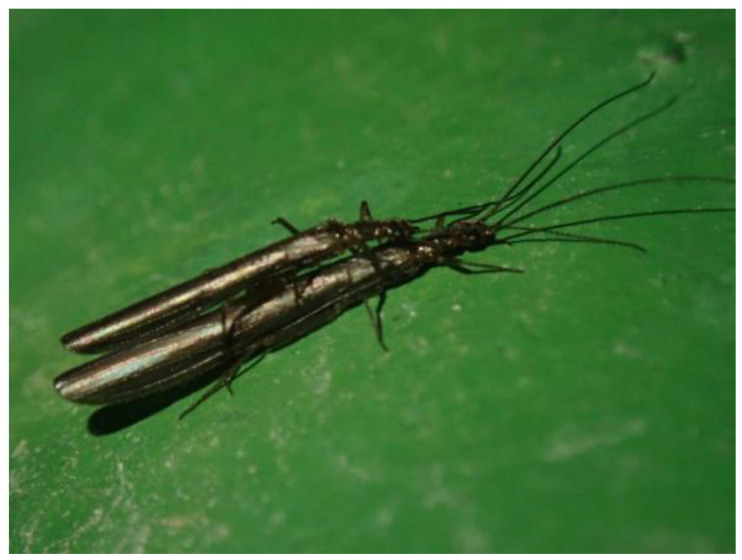
*Rhopalopsole sinensis* Yang and Yang, 1993, the mating adults (photo by Xin-Xing Luo).

*Rhopalopsole sinensis* Yang and Yang, 1993 (Figure 1) belongs to the plecopteran family Leuctridae. It is the most common species of the genus *Rhopalopsole* in China and is widely distributed in the areas south of the Qinling Mountains and east of the Tibetan Plateau [14,15]. The classic method of studying species is morphological taxonomy, but the simple use of morphological methods may underestimate the true biological diversity within a species. Generally, the external morphology of cryptic species is relatively conservative in evolution (Bickford et al., 2007). At present, research on Plecoptera mainly focuses on the traditional taxonomy and phylogenetic relationships of high taxonomic groups [6,16,17,18]. However, few studies [18,19,20] have combined the infraspecific genetic diversity, population genetic structure, molecular phylogeny and population dynamics.

Mutation, natural selection, genetic drift, and gene flow are the main evolutionary forces affecting phylogeography and evolutionary history within species [21,22,23,24]. Mitochondrial DNA (mtDNA) has the characteristics of fast base substitution rate, simple structure, is inherited maternally, has almost no recombination, exhibits moderate gene evolution speed, and has established primers for amplification and sequencing [25,26,27]. Owing to its relatively high degree of variation, the *cytochrome oxidase subunit I* (*COI*) gene encoded by mtDNA has been used to study population genetic polymorphisms in various insect species. Mitochondrial genes can be used to solve the phylogenetic problems of infraspecific category, but introgression may not be ruled out [26]. Thus, if the analysis of nuclear and mitochondrial genes is combined, the molecular phylogenetic results will be more reliable. Among numerous nuclear genes, the *18S rDNA* gene and *ITS2* gene are widely used in insect phylogenetic research, especially to identify the existence of synonyms or cryptic species, and describe the intraspecific geographical differentiation [28,29,30,31,32,33,34,35].

**Figure 2:**
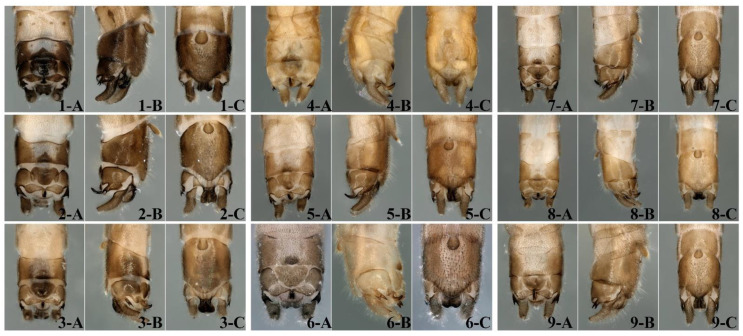
*Rhopalopsole sinensis* Yang and Yang, 1993. Terminal abdominal segments of males from different Chinese provinces. 1: Anhui; 2. Zhejiang; 3. Hunan; 4. Guangxi; 5. Fujian; 6. Guizhou; 7. Sichuan; 8. Guangdong; 9. Shaanxi. (**A**) Dorsal view; (**B**) lateral view; (**C**) ventral view.

Chen [36] found that the intraspecific genetic distance between Fujian’s and Guangxi’s specimens of *R. sinensis* was high (15%), suggesting distinct geographic populations or unrecognized cryptic species for this widespread taxon in southern China. However, when we examined specimens of *R. sinensis* from different geographical populations, we [14,37] did not find any morphological differences (Figure 2). Here, we use molecular data to support the geographical distribution and faunal regions of *R. sinensis* and determine the existence of cryptic species. This study sequenced the *18S, COI*, and *ITS2* genes on recently collected specimens of *R. sinensis* to determine the genetic structure of 9 geographically distinct populations using Bayesian inference (BI) and maximum likelihood (ML) methods to construct species level phylogenetic trees.

## 2. Materials and Methods

### 2.1. Specimen Preparation

Most of the adults of *R. sinensis* live in the bushes or small trees near clean and cold streams. Due to their weak flight ability, we used a net to shake the twigs or branches, and the adults fell into the insect net for collection. The adults of *Rhopalopsole* show phototaxis, so they can also be collected by a light trapping method. Specimens were preserved in 75% ethanol. Male terminalia were cleared in 10% NaOH solution. All specimens used in this study are deposited in the Insect Collection of Yangzhou University (ICYZU), Jiangsu Province, China.

Sampling localities include (abbreviations of province or autonomous region of China): Anhui (AH), Fujian (FJ), Guangdong (GD), Guangxi (GX), Guizhou (GZ), Hunan (HN), Sichuan (SC), Shaanxi (SX), Zhejiang (ZJ).

### 2.2. Observation and Description

Morphological details were examined using a Leica MZAPO microscope (Leica Microsystems Ltd., Heerbrugg, Switzerland). Color illustrations were taken with a KEYENCE VHX-5000 (Keyence, Ōsaka, Japan). Our concept of *R. sinensis* are defined by a series of morphological characteristics, including the long hairs on the antennae; tergum 9 mostly sclerotized, with a semicircular and sclerotized area in the middle of the posterior margin; sternum 9 basally with flabelliform vesicle bearing dense hairs, apically with semiellipitcal subgenital plate; tergum 10 with lateral processes sclerotized and each formed a bicuspid process in lateral view; the middle sclerotized part is wide and trapezoid, and the two sides are short and thick; the length of the posterior transverse sclerites is nearly equal to the width of tergum 10; cercus hairy and upcurved, with a small spine; epiproct is small and forming an upcurved hook, of which the top becomes tapered (Figure 1 and Figure 2).

### 2.3. DNA Extraction, Amplification and Sequencing

The specimens used for molecular analyses were collected from 9 different Provinces in China (Tables S1–S3). Genomic DNA was extracted using AxyPrep™ Multisource Genomic DNA Kit (Axygen, New York, NY, USA), following the manufacturer’s instruction.

For the nuclear markers, 636–661 bp, 353–370 bp and 663 bp fragments of *18S*, *ITS2* and *COI* respectively, were amplified from individuals using the primer pairs as listed in Table 1. The primers used to amplify these three fragments are also used for the sequencing reactions. The polymerase chain reaction (PCR) reactions were conducted with protocols described in Yang and Du [32]. Amplification products were purified and sequenced by Biozeron Biotech Co. (Shanghai, China) in both directions using the same primers as above. The sequences were submitted to National Center for Biotechnology Information (NCBI), a branch of the USA National Institutes of Health (Tables S1–S3).

### 2.4. Sequential Analysis

We utilized CLUSTAL X 1.83 [38] to manually align the *COI, 18S*, *ITS2* sequences. DNASP v. 5.0 [39] was used to calculate the number of haplotypes, nucleotide diversity (π), haplotype diversity (Hd), as well as average number of nucleotide differences (K).

### 2.5. Population Relationship Analyses

The genetic distance and maximum likelihood (ML) analyses were computed using the program MEGA v. 7.0. The parameters of ML analysis are as follows—test of phylogeny: bootstrap method with 1000 bootstrap replications; substitution type: nucleotide; model/method: Kimura 2-parameter model; rates among sites: uniform rates; gaps/missing data treatment: complete deletion; ML heuristic method: nearest-neighbor-interchange (NNI); initial tree for ML: make initial tree automatically (Default-NJ/BioNJ); branch swap filter: none.

MrBayes v. 3.1.2 was utilized to generate the topology for the Bayesian inference (BI) analysis, the working procedure is as follows: execute XXX.nex—lset nst = 6 rate = invgamma—showmodel—mcmc ngen = 100,000 samplefreq = 100—If the value is greater than 0.01, continue; if it is less than 0.01, enter: sump burnin = 250 (total running generation/sampling frequency then divided by 4)—sumt burnin = 250

We also constructed split networks to reveal relationships among haplotypes using the Flexus Network v. 4.6 program [40].

### 2.6. Population Genetic Structure Analyses

F-statistic values (*F*_ST_; differentiation index) and Gene Flow (*Nm*) of 3 genes between populations were calculated based on ARLEQUIN v. 3.5 [41]. Repeat sampling 10,000 times to test the significance of *F*_ST_. Perform molecular analysis of variance (AMOVA) on 9 geographical populations of *R. sinensis*, estimate the distribution of genetic variation, and conduct significance analysis after random repeated sampling of 10,000 times. The Panmixia population hypothesis was conducted through Exact test of population differentiation.

### 2.7. Demographic History Analyses

The demographic history of *R. sinensis* populations in China was studied using mismatch distributions in ARLEQUIN v. 3.5 [41]. Harpending’s raggedness (HR) index was also calculated in ARLEQUIN. Tajima’s *D* and Fu’s *Fs* tests were used to test for neutrality. All parameters were evaluated based on 1000 bootstrap replicates.

### 2.8. Niche Model Analysis

We used ecological niche modeling to predict the geographic distribution of climatically suitable habitats for *R. sinensis* in China. MAXENT [42] was utilized to analyze whether climatic stability as well as current and past climate conditions are responsible for observed patterns of *R. sinensis* genetic diversity and population structure. We obtained bioclimatic data layers with 2.5-m spatial resolution for the last interglacial (LIG), last glacial maximum (LGM), and current conditions from the WorldClim database (http://worldclim.org/current.htm; accessed on 2 November 2023) [43]. Nineteen bioclimatic variables (Bio1–19) were also obtained from the database WorldClim to identify potential key variables that infer their distribution. We removed seven variables (Bio6, Bio7, Bio9, Bio10, Bio11, Bio16 and Bio17) due to their significant correlation (jrj > 0.8) as evaluated by the Pearson’s correlation coefficient calculation computed using ENMTOOLS [44]. To avoid spatial autocorrelation in MAXENT models, we estimated the best values of the regularization multiplier (RM) and the feature classes (FCs) in ENMEVAL package [45]. We ran the models with combinations of RM between 1 and 4 with steps of 0.5 and the FCs (L = Linear, Q = Quadratic, H = Hinge, P = Product). We set the default of 10,000 random background points. The geographical distribution data of *R. sinensis* in China was obtained from our sampling in this study (Tables S1–S3). We evaluated the relevance of the selection of the threshold in Maxent using two different options: minimum training presence and tenth percentile training presence. The random test percentage was set to 25%, and the Jackknife procedure was used to estimate the contribution of each variable based on performance of the model. The area under the curve (AUC) value was calculated for model validation, and AUC reflects the model’s ability to distinguish between present records and random background points. AUC values ranged from 0.5 (not different from a randomly-selected predictive distribution) to 1.0 (with perfect predictive ability). Models were generated in ASCII format and exported directly to ArcGIS platform (http://www.esri.com/software/arcgis; accessed on 27 July 2023). Two thresholds were selected for Maxent models: the minimum training presence and the tenth percentile training presence. The final map was visualized and processed using the ArcGIS platform.

## 3. Results

### 3.1. High Level of Genetic Diversity

The result shows in the Tables S4–S6: the genetic differences in *COI* gene sequences, *18S* gene sequences, and *ITS2* gene sequences among the same geographical population are between 0–2.8%, 0–0.9%, and 0–3%, respectively. Therefore, it can be determined that the same geographical population belongs to the same species.

### 3.2. COI Gene

The length of the *COI* gene is 663 bp, and 169 bp were polymorphic between populations. A total of 18 haplotypes were identified for the *COI* gene between nine populations. In particular, 15 were unique haplotypes present in only one individual, while the other 3 haplotypes were present in at least two or more individuals. We examined the polymorphisms, among those sites, 12 are singleton variable sites, and 157 are parsimony informative sites. The nine populations in China showed different levels of genetic variation, with most exhibiting high levels of variation. The mean haplotype diversity (Hd) throughout all populations was 0.972. The mean nucleotide diversity (π) was 0.09678 and the average number of nucleotide differences (K) was 63.972 (Table 2).

### 3.3. 18S Gene

The length of the *18S* gene is 636–661 bp, and 98 bp were polymorphic between populations. A total of 12 haplotypes were identified for the *18S* gene. In particular, nine of them were unique haplotypes present in only one individual, while the other three haplotypes were present in at least two or more individuals. We examined the polymorphisms, and among those sites, 20 are singleton variable sites, while 78 are parsimony informative sites. The seven populations in China showed different levels of genetic variation, with most exhibiting high levels of variation. The mean haplotype diversity (Hd) throughout all populations was 0.958. The mean nucleotide diversity (π) was 0.04645 and the average number of nucleotide differences (K) was 29.683 (Table 2).

### 3.4. ITS2 Gene

The length of the *ITS2* gene is 353–370 bp, 75 bp were polymorphic between populations. A total of 21 haplotypes were identified for the *ITS2* gene. In particular, 19 of them were unique haplotypes present in only one individual, while the other two haplotypes were present in at least two or more individuals. We examined the polymorphisms, among those sites, 20 are singleton variable sites, and 55 are parsimony informative sites. The 9 populations in China showed different levels of genetic variation, with most exhibiting high levels of variation. The mean haplotype diversity (Hd) throughout all populations was 0.992. The mean nucleotide diversity (π) was 0.06082 and the average number of nucleotide differences (K) was 20.984 (Table 2). These indexes suggest that *R. sinensis* exhibited high level of genetic diversity.

### 3.5. Population Genetic Structure

**Figure 3:**
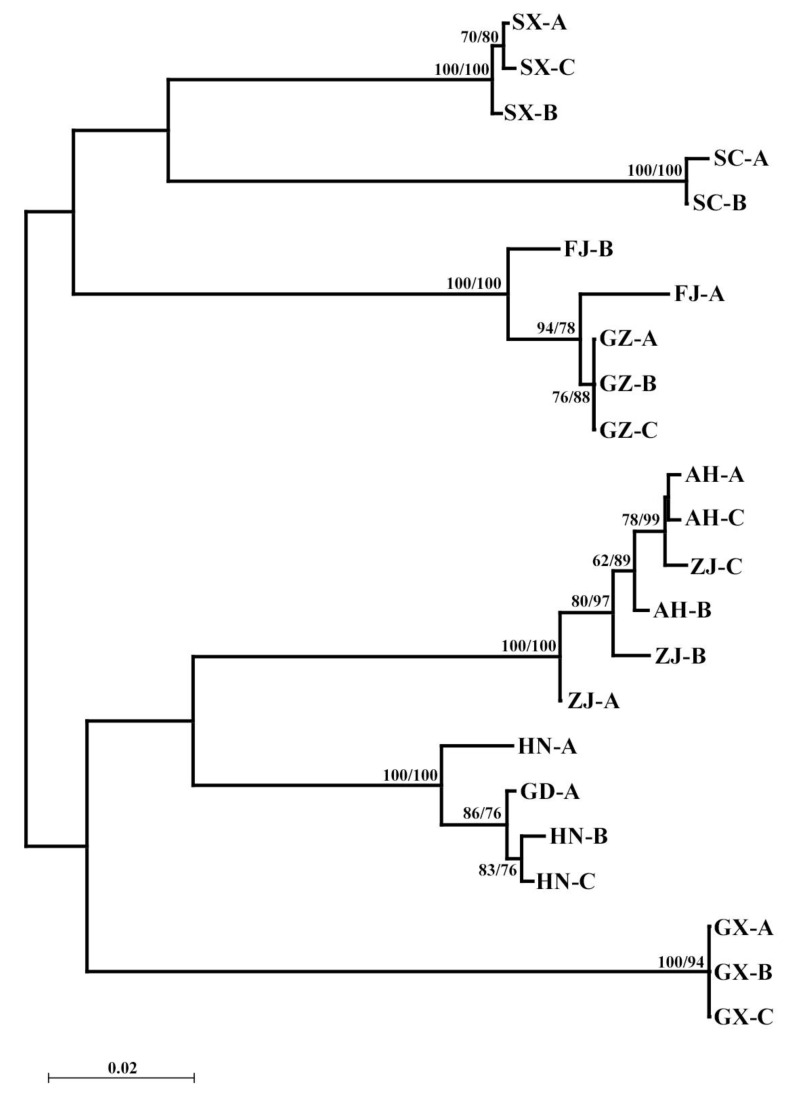
Phylogenetic trees of *COI* gene. Phylogenetic relationships among nine geo-populations of *R. sinensis* based on maximum likelihood (ML) analysis and Bayesian inference (BI) tree (numbers at the nodes are ML bootstrap values (**left**) and Bayesian posterior probabilities (**right**); only nodal support values of >60% are depicted on the tree). The scale bar represents the rate of base substitutions.

The maximum likelihood tree and the Bayesian tree showed the results in Figure 3. It can be found that different populations of *R. sinensis* are roughly clustered into two major groups, with a confidence level of over 60%. Among them, Guangxi, Huanan, Guangdong, Zhangjiang, and Anhui are grouped into a clade; Guizhou, Fujian, Sichuan, and Shanxi are grouped into another clade. This result is consistent with the genetic distance between geographical populations (Tables S4–S6). The results of AMOVA demonstrated that there were genetic variations among and within nine different geographical populations of *R. sinensis*, with 93.35% variation between populations and 6.65% variation within populations (Tables S7–S9). The genetic differences between individuals are significantly higher than those within populations. Data analysis shows that only three sets of data > 1 for gene flow among different geographical populations, with an overall fixed coefficient *F*_ST_ of 0.93347, indicating significant genetic differentiation among different geographical populations of *R. sinensis*.

**Figure 4:**
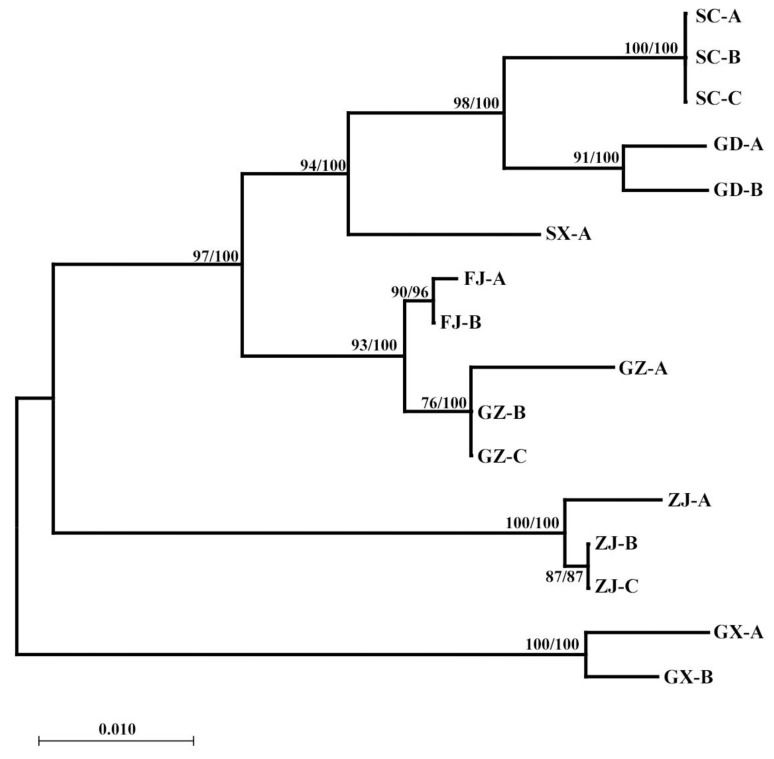
Phylogenetic trees of *18S* gene. Phylogenetic relationships among seven geo-populations of *R. sinensis* based on maximum likelihood (ML) analysis and Bayesian inference (BI) tree (numbers at the nodes are ML bootstrap values (**left**) and Bayesian posterior probabilities (**right**); only nodal support values of >60% are depicted on the tree). The scale bar represents the rate of base substitutions.

**Figure 5:**
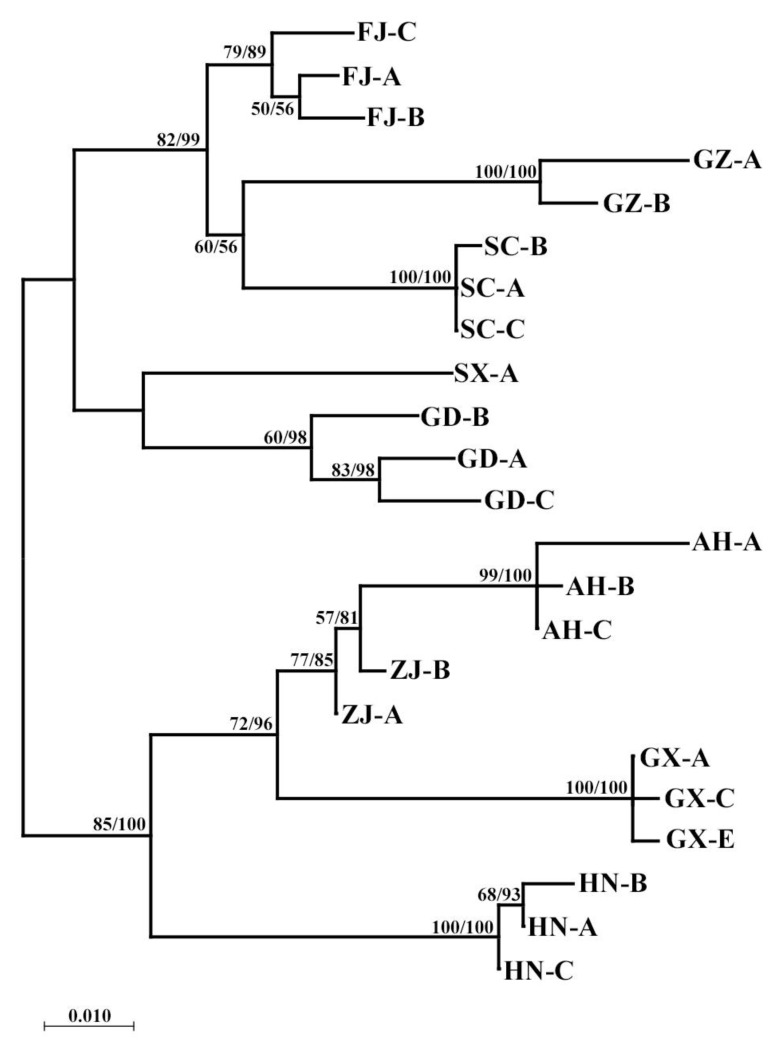
Phylogenetic trees of *ITS2* gene. Phylogenetic relationships among nine geo-populations of *R. sinensis* based on maximum likelihood (ML) analysis and Bayesian inference (BI) tree (numbers at the nodes are ML bootstrap values (**left**) and Bayesian posterior probabilities (**right**); only nodal support values of >60% are depicted on the tree). The scale bar represents the rate of base substitutions.

The same methods were used to construct the *18S* gene phylogenetic tree of 7 geographical populations of *R. sinensis*. Based on the results in Figure 4, the clustering pattern is Guangxi + (Zhejiang + (Fujian + Guizhou + Shaanxi + Guangdong + Sichuan)). This result is consistent with the genetic distance between geographical populations (Table S5). The results of AMOVA showed that there were genetic variations among and within nine different geographical populations of *R. sinensis*, with 89.89% variation between populations and 10.11% variation within populations (Tables S7–S9); the genetic differences between species are significantly higher than those within populations. The *Nm* values between the seven geographical populations of *R. sinensis* are all <1, with an overall fixed coefficient *F*_ST_ of 0.89893. Based on the results in Figure 5, the clustering pattern is (Hunan + Guangxi + Zhejiang + Anhui) + (Guangdong + Shaanxi + Sichuan + Guizhou + Fujian). This result is consistent with the genetic distance between geographical populations. The AMOVA showed that there were genetic variations among and within nine different geographical populations of *R. sinensis*, with 85.93% variation between populations and 14.07% variation within populations. The genetic differences between species are significantly higher than those within populations. The *Nm* values among the 9 geographical populations of *R. sinensis* are all <1, with an overall fixed coefficient *F*_ST_ of 0.85934 (Tables S10–S15). All results indicate that there is significant genetic differentiation among different geographical populations of *R. sinensis*. The Exact test results showed that there was no significant difference in the distribution frequency of the haplotypes among the nine geographical populations of *R. sinensis* (Tables S16–S18), which was consistent with the hypothesis that it was a Panmixia population, indicating that reproductive isolation has not yet formed among different geographical populations of *R. sinensis*, so *R. sinensis* has not yet formed cryptic species.

### 3.6. Demographic History

**Figure 6:**
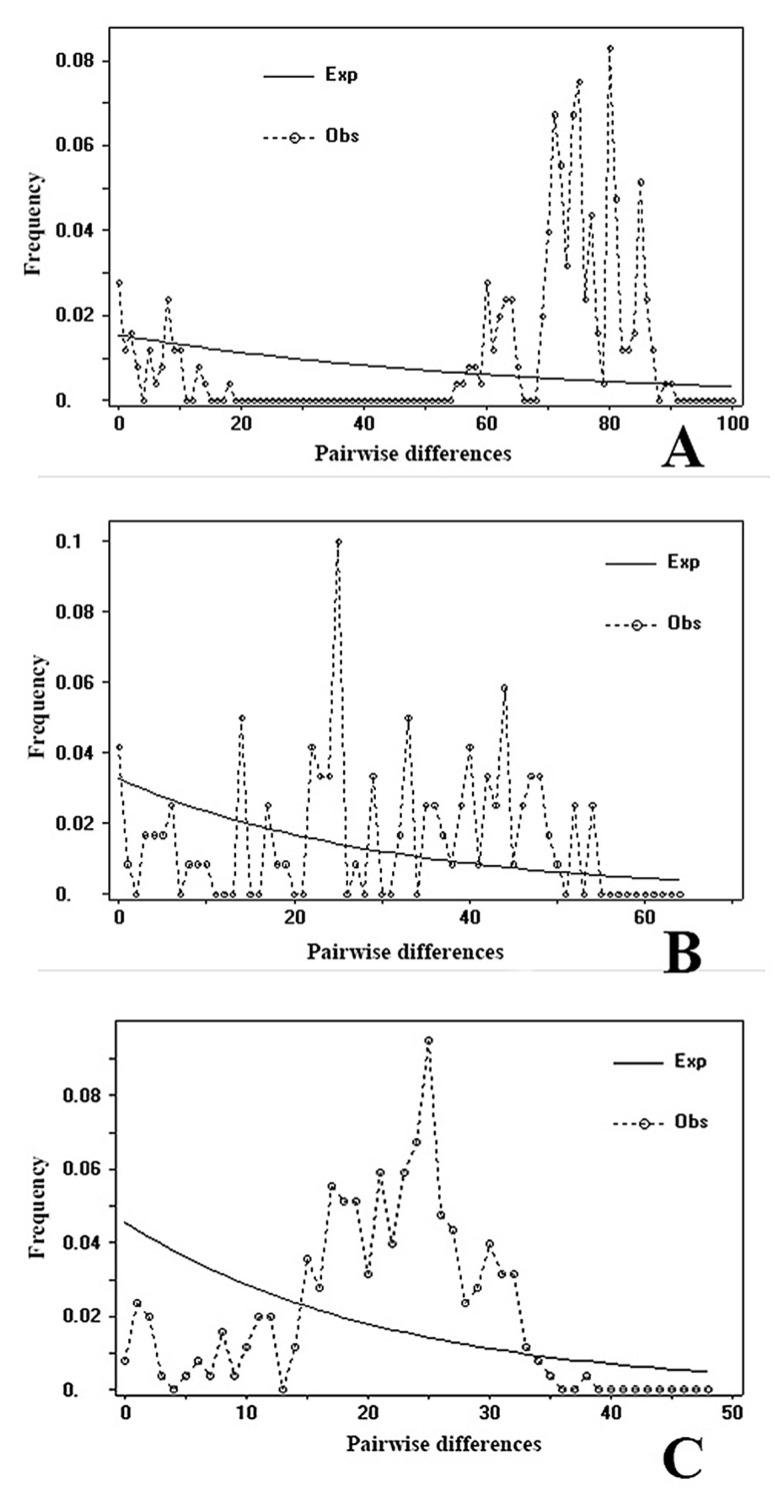
Mismatch distribution of (**A**): *COI* haplotypes; (**B**): *18S* haplotypes; (**C**): *ITS2* haplotypes in *R. sinensis* populations.

To uncover the demographic history of *R. sinensis* in China, neutrality tests were conducted using Tajima’s *D* and Fu’s *Fs* statistics based on the three genes sequenced. The Tajima’s *D* values and Fu’s *Fs* statistic of *COI* gene for all the samples were positive (*D* = 0.70655; *Fs* = 2.361); the Tajima’s *D* values of *18S* gene for all the samples were negative (*D* = −0.10697), while Fu’s *Fs* statistic was positive (*Fs* = 1.693); the Tajima’s *D* values of *ITS2* gene for all the samples were negative (*D* = −0.51755; *Fs* = 2–4.771), but all the results are not significant (*p* > 0.10) (Table 3). The multimodal mismatch distribution of all *R. sinensis* samples (Figure 6) may indicate that the *R. sinensis* populations in China fit a neutral evolution model or our samples cover several divergent populations based on our analyses.

## 4. Conclusions

The results of genetic structure analysis show that the nine geographical populations of *R. sinensis* have significant genetic differentiation, but the results of Exact tests do not support the formation of reproductive isolation between different geographical populations, indicating that *R. sinensis* has not yet formed cryptic.

The establishment of the *18S* gene and *ITS2* gene supports the population of (Guangdong + Shaanxi + Sichuan + Guizhou + Fujian) as independent branches, while the population of (Anhui + Zhejiang + Hunan) is another branch. The establishment of *COI* gene tree supports (Shaanxi + Sichuan + Guizhou + Fujian) as one branch, while the other independent branch is composed of (Anhui + Zhejiang + Guangxi + Hunan + Guangdong) groups. This result is slightly different from the phylogenetic trees based on the *18S* gene and *ITS2* gene.

## 5. Discussion

### 5.1. Molecular Evolution

In summary, the *COI* gene, *18S* gene and *ITS2* gene can be used to study the genetic differentiation of the widely distributed species *R. sinensis*. The differences in these results may be related to the number of specimens sequenced, the selection of tree building methods or models, all of which can affect the topological structure of the phylogenetic trees [46]. In the future, investigating the phylogenetic relationships of species through comparative genomics may be more accurate than morphological assessment alone, and reflect the actual evolution of a species. In the future, more species samples and genetic data will be needed to estimate that if *R. sinensis* has the increased probability to form a cryptic species due to significant genetic differentiation and low frequency of gene exchange [47].

**Figure 7:**
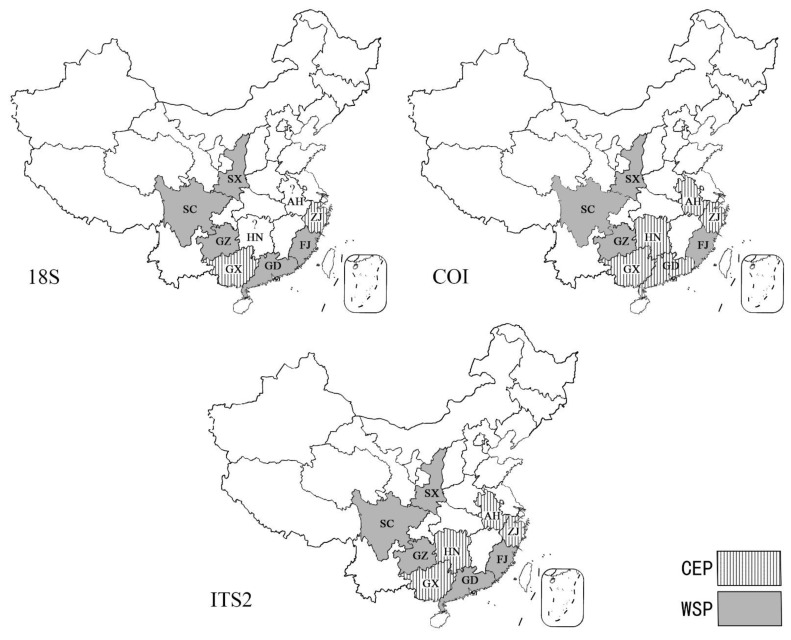
The distribution map of the Central-Eastern and Western-Southern Populations (CEP and WSP) of *R. sinensis* populations in China.

In these phylogenetic trees, these populations from different regions were divided into two large branches (Figure 7), which were called Central-Eastern Populations (CEP, Anhui + Zhejiang + Guangxi +Hunan) and Western-Southern Populations (WSP, Sichuan, Shaanxi, Guizhou, Fujian, Guangdong). In most cases, the clustering of CEP and WSP had a stable correspondence with their regions, except for the Guangdong population, which was classified as CEP in the *COI* analysis. *COI* is a mitochondrial gene with stable maternal inheritance characteristics [48], and nuclear genes may change with gene flow between populations [49]. The results of the nuclear gene analysis in the Guangdong population were inconsistent with the *COI*, which was probably due to the fact that the region was located in the transition zone between the Pearl River Basin and the Yangtze River Basin, and the two populations of *R. sinensis* were likely to have had gene exchange in the local area before, resulting in the convergence of some individuals at the nuclear gene level. Another possible reason is that the direction and rate of evolution of the local populations in nuclear and mitochondrial genes are inherently inconsistent. The results of this paper only reveal the existence of such inconsistencies, but there is too little material available to quantitatively analyze the size and distribution boundaries of various groups. Therefore, there is still a need for larger surveys and increased sampling in the future.

It is important to emphasize that the populations in Fujian and Guangdong are far from the western provinces such as Sichuan, but they belong to the same WSP, while the Hunan and Guangxi populations are geographically closest to Guizhou, Sichuan and Shaanxi, but belong to two different large populations. It is necessary to combine zoogeographical data with these genetic data to explain why the boundaries of the two populations were formed in these areas.

### 5.2. The Origin and Immigration

**Figure 8:**
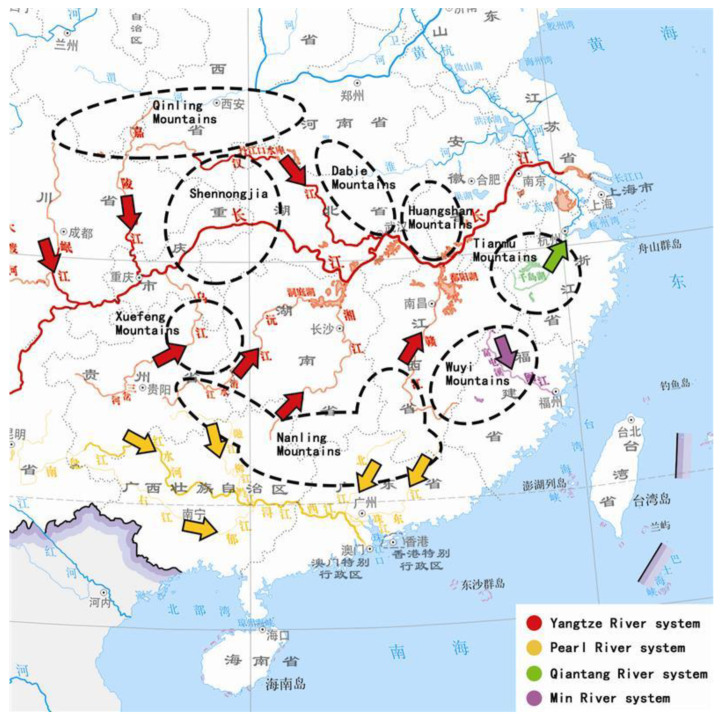
Map of the main water system of the middle and lower reaches of the Yangtze River and the Pearl River (the flow direction of the first-order tributaries is marked with arrows). Map revised by www.tianditu.gov.cn, accessed on 10 February 2025.

Regarding the origin and dispersal of Plecoptera, it is now widely accepted that the direction of dispersal of the suborder Euholognatha is “from north to south and from west to east” [5]. In China, many studies have highlighted that the distribution of stonefly fauna is closely related to the health of the water system [50,51,52], but this does not mean that all populations of a taxa in the same river system (or basin) are homologous or most closely related. Due to the complexity of the topography of Chinese mainland, the well-known Yangtze River, Yellow River and other river basins have long been divided into different areas by multiple mountain ranges (Figure 8), and the formation of insect fauna and population distribution have been affected and limited to varying degrees [13]. We believe that the movement of the mountain range may be more important than the simple concept of “water system” in the distribution pattern of stoneflies, because the growth of the stonefly is inseparable from water, and the direction of water is always inseparable from the influence of topography.

**Figure 9:**
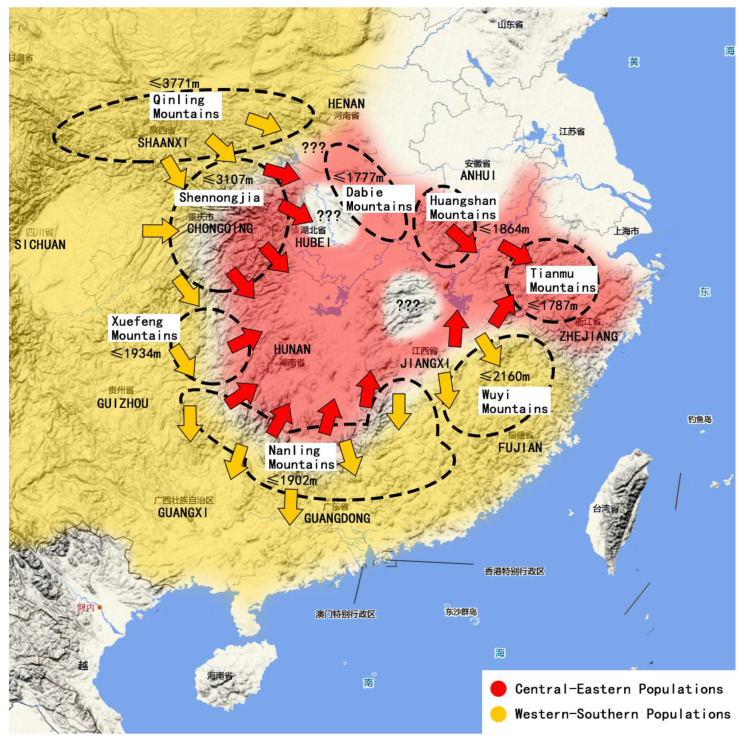
The hypothetical immigration of *R. sinensis* in southeastern China (the migrating direction of populations are marked with arrows). Map revised by www.tianditu.gov.cn, accessed on 10 February 2025.

According to recent research, the formation of the middle and lower reaches of the modern Yangtze River is something that happened in the last 2 million years [53,54,55]. The formation of the “three-rung ladder-like topography“ on the Chinese mainland, and the Qinling to Shennongjia area, are the junction of China’s north-south directions and have altitudes of more than 3000 m. The elevation from these areas to the east and south is getting lower and lower: the highest altitude is higher in the Xuefeng Mountain, Nanling and Wuyi Mountains, about 1900–2200 m, and in the eastern areas of Dabie Mountain, Huangshan Mountain and Tianmu Mountain, the highest altitude is no more than 1800 m (Figure 9), while the elevation of the sampling points of *R. sinensis* is often only a few hundred meters. Based on our sampling experience, most of the habitats of *R. sinensis* are located in the small tributaries of the major mountain ranges in the Yangtze River basin, rather than the wide Yangtze River itself and its first-order tributaries–there is currently no evidence that Chinese stoneflies can naturally climb over large areas of high mountains or disperse to their source (Tibetan Plateau/Yunnan-Guizhou Plateau) through the mainstream/primary tributaries of the Yangtze or Pearl rivers. Adults of Plecoptera are known to be weak in flight [56] and larvae are very dependent on water quality. For aquatic insects, these mountains are effectively islands. The natural dispersal of populations, as well as genetic exchange with populations in neighbouring regions, can be blocked by intervening areas of unsuitable habitat, even though these areas are not far apart. However, Zwick [56] also emphasizes that “the current distribution pattern is the result of speciation and extinction events, not a dispersal process”, and we cannot completely rule out the existence of potential (or still maintaining) genetic exchange events in stoneflies between these regions. Since Heiman and Knight [57] have previously demonstrated that the eggs of stoneflies are resistant to high temperatures, and that some species have the biological characteristics of dormancy or diapause with eggs or larvae [58], we do not rule out that Chinese stoneflies can be randomly carried to the lower reaches of certain river systems with seasonal large-scale water movements (such as floods caused by the rainy season), or migrate back and forth in streams between neighboring mountain ranges.

Based on the results of molecular and geographical analysis, we believe that *R. sinensis* originated in the western part of China (possibly from the western Qinling Mountains to the northern Hengduan Mountains) and migrated to the mountains along the southeast coast with the formation and eastward migration of the Yangtze River. The two major populations of *R. sinensis* crossed the Yangtze River and Pearl River basins, but there was no obvious correlation in the horizontal distribution. Guangdong-Guangxi may be a transition zone between these two populations, roughly bounded by the Nanling Mountains (southern Hunan, northeastern Guangxi, and northern Guangdong). It is worth noting that there are other species of stoneflies that are widespread in East Asia, such as *Amphinemura chui* (Wu, 1935), *Paraleuctra orientalis* (Chu, 1928), *Togoperla canilimbata* (Enderlein, 1909), *T. perpicta* Klapálek, 1921 and *Hemacroneuria violacea* Enderlein, 1909 etc. (that can also be distributed along Zhejiang, Fujian, Guangdong, Guangxi and even Vietnamese routes, while they have not been recorded in the northern part of this route. This indicates at least to a certain extent the uniqueness of the fauna of the Shennongjia to Xuefeng Mountains, the south of Qinling Mountains, and the north of Wuyi to Nanling Mountains, as well as the continuity of the distribution of some genera and species of Plecoptera in the “East Asian Rim Coast” region. Given increasing human activity and urbanization, these mountainous areas should be considered the common (and the last) refugia of the stoneflies in southern China. Our hypothesis is that these stonefly populations in any one high-altitude mountain may radiate out into the surrounding low-altitude streams, and create a series of separate populations that share the same/similar origin but do not geographically meet. However, we do not rule out that these dispersed populations may have occasional and limited encounters to have some genetic exchange due to outstanding environmental factors (such as potential caves or underground rivers, seasonal flooding, etc.).

## Figures and Tables

**Table 1:** Primer sequences of three genes.

Primer	Sequence
*COⅠ*-LCO1490	GGTCAACAAATCATAAAGATATTGG
*COⅠ*-HCO2198	TAAACTTCAGGGTGACCAAAAAATCA
*18S*-a0.7	ATTAAAGTTGTTGCGGTT
*18S*-b2.9	TATCTGATCGCCTTCGAACCTCT
*ITS2*-Forward	GCATCGATGAAG AACGCAGC
*ITS2*-Reverse	TCCTCCGCTTATTGATATGC

**Table 2:** Parameters of genetic diversity of *R. sinensis* based on three genes.

Gene Name	Sample Size	Number of Polymorphic Sites	Average number of Nucleotide Differences (*K*)	Haplotype Number	Haplotype Diversity (Hd)	Mean Nucleotide Diversity (*π*)
*COI*	23	169	63.972	18	0.972	0.09678
*18S*	16	98	29.683	12	0.958	0.04645
*ITS2*	23	75	20.984	21	0.992	0.06082

**Table 3:** The results of neutrality tests.

Gene Name	Tajima’s *D* Test	Fu’s *Fs* Test
*COⅠ*	0.70655 (*p* > 0.10)	2.361 (*p* > 0.10)
*18S*	−0.10697 (*p* > 0.10)	1.639 (*p* > 0.10)
*ITS2*	−0.51755 (*p* > 0.10)	−4.771 (*p* > 0.10)

## Data Availability

The data that support the findings of this study are openly available in NCBI: GenBank accession nos. of *COI*: OQ909068-OQ909072 and OQ913470-OQ913487. GenBank accession nos. of *18S*: OQ911553-OQ911568. GenBank accession nos. of *ITS2*: OQ911530-OQ911552.

## Supplementary Materials

The following supporting information can be downloaded at: https://www.mdpi.com/article/10.3390/insects16030244/s1. Table S1. Sample information for the 18S gene of *R. sinensis*. Table S2. Sample information for the COⅠ gene of *R. sinensis*. Table S3. Sample information for the ITS2 gene of *R. sinensis*. Table S4. Genetic Distance (%), COⅠ. Table S5. Genetic Distance (%), 18S. Table S6. Genetic Distance (%), ITS2. Table S7. Analysis of molecular variance (AMOVA) of mitochondrial DNA COI for 23 individuals in 9 *R. sinensis* populations. Table S8. Analysis of molecular variance (AMOVA) of 18S rDNA for 17 individuals in 8 *R. sinensis* populations. Table S9. Analysis of molecular variance (AMOVA) of ITS2 for 23 individuals in 9 *R. sinensis* populations. Table S10. The Fst value and genetic flow based on COI gene among 9 geo-populations of *R. sinensis*. Table S11. The Fst-p value and Exact test results based on COI gene among 9 geo-populations of *R. sinensis*. Table S12. The Fst value and genetic flow based on ITS2 gene among 9 geo-populations of *R. sinensis*. Table S13. The Fst-p value and Exact test results based on ITS2 gene among 9 geo-populations of *R. sinensis*. Table S14. The Fst value and genetic flow based on 18S gene among 7 geo-populations of *R. sinensis*. Table S15. The Fst-p value and Exact test results based on 18S gene among 7 geo-populations of *R. sinensis*. Table S16. Haplotype distribution (frequencies) through *R. sinensis* populations. Table S17. Haplotype distribution (frequencies) through *R. sinensis* populations. Table S18. Haplotype distribution (frequencies) through *R. sinensis* populations.
