# Supplementary material for: Gone with Water or Mountain: The Population Genetic Diversity of Rhopalopsole sinensis Yang and Yang, 1993 in China
Source: Insects. 2025 Feb 26;16(3):244. doi: 10.3390/insects16030244 (PMC11942876; doi:10.3390/insects16030244)
Supplement: Supplementary file 1 [file insects-16-00244-s001.zip › insects-3474295-supplementary.pdf]

Table S1. Sample information for the 18S gene of *R. sinensis*

Number	Collection Locations	code	Collecting date (year/month)	Latitude/longitude	GenBank No.
1	Zhejiang, Lishui	ZJA	2021/03	28.48N/119.90E	OQ911561
2	Zhejiang, Lishui	ZJB	2021/03	28.48N/119.90E	OQ911562
3	Zhejiang, Lishui	ZJC	2021/03	28.48N/119.90E	OQ911563
4	Guangxi, Guilin	GXA	2020/08	25.90N/110.45E	OQ911564
5	Guangxi, Guilin	GXB	2020/08	25.90N/110.45E	OQ911565
6	Fujian, Wuyishan	FJA	2021/05	30.47N/103.13E	OQ911559
7	Fujian, Wuyishan	FJB	2021/05	30.47N/103.13E	OQ911560
8	Guizhou, Zunyi	GZA	2011/11	28.28N/107.17E	OQ911566
9	Guizhou, Zunyi	GZB	2011/11	28.28N/107.17E	OQ911567
10	Guizhou, Zunyi	GZC	2011/11	28.28N/107.17E	OQ911568
11	Sichuan, Ya’an	SCA	2021/06	30.20N/103.05E	OQ911555
12	Sichuan, Ya’an	SCB	2021/06	30.20N/103.05E	OQ911556
13	Sichuan, Ya’an	SCC	2021/06	30.20N/103.05E	OQ911557
14	Guangdong, Nanling	GDA	2021/05	24.90N/113.03E	OQ911553
15	Guangdong, Nanling	GDB	2021/05	24.90N/113.03E	OQ911554
16	Shaanxi, Yan’an	SXA	2019/07		OQ911558

Table S2. Sample information for the COI gene of *R. sinensis*

Number	Collection Locations	code	Collecting date (year/month)	Latitude/longitude	GenBank No.
1	Anhui, Chizhou	AHA	2021/05	30.47N/117.80E	OQ913473
2	Anhui, Chizhou	AHB	2021/05	30.47N/117.80E	OQ913474
3	Anhui, Chizhou	AHC	2021/05	30.47N/117.80E	OQ913475

4	Zhejiang, Lishui	ZJA	2021/03	28.48N/119.90E	OQ913476
5	Zhejiang, Lishui	ZJB	2021/03	28.48N/119.90E	OQ913477
6	Zhejiang, Lishui	ZJC	2021/03	28.48N/119.90E	OQ913478
7	Hunan, Chenzhou	HNA	2020/09	24.97N/112.90E	OQ913482
8	Hunan, Chenzhou	HNB	2020/09	24.97N/112.90E	OQ913483
9	Hunan, Chenzhou	HNC	2020/09	24.97N/112.90E	OQ913484
10	Guangxi, Guilin	GXA	2020/08	25.90N/110.45E	OQ913479
11	Guangxi, Guilin	GXB	2020/08	25.90N/110.45E	OQ913480
12	Guangxi, Guilin	GXC	2020/08	25.90N/110.45E	OQ913481
13	Fujian, Wuyishan	FJA	2021/05	30.47N/103.13E	OQ913471
14	Fujian, Wuyishan	FJB	2021/05	30.47N/103.13E	OQ913472
15	Guizhou, Zunyi	GZA	2011/11	28.28N/107.17E	OQ913485
16	Guizhou, Zunyi	GZB	2011/11	28.28N/107.17E	OQ913486
17	Guizhou, Zunyi	GZC	2011/11	28.28N/107.17E	OQ913487
18	Sichuan, Ya’an	SCA	2021/06	30.20N/103.05E	OQ909068
19	Sichuan, Ya’an	SCB	2021/06	30.20N/103.05E	OQ909069
20	Guangdong, Nanling	GDA	2021/05	24.90N/113.03E	OQ913470
21	Shaanxi, Yan’an	SXA	2019/07		OQ909070
22	Shaanxi, Yan’an	SXB	2019/07		OQ909071
23	Shaanxi, Yan’an	SXC	2019/07		OQ909072

Table S3. Sample information for the ITS2 gene of *R. sinensis*

Number	Collection Locations	code	Collecting date (year/month)	Latitude/longitude	GenBank No.
1	Anhui, Chizhou	AHA	2021/05	30.47N/117.80E	OQ911540
2	Anhui, Chizhou	AHB	2021/05	30.47N/117.80E	OQ911541
3	Anhui, Chizhou	AHC	2021/05	30.47N/117.80E	OQ911542
4	Zhejiang, Lishui	ZJA	2021/03	28.48N/119.90E	OQ911543
5	Zhejiang, Lishui	ZJB	2021/03	28.48N/119.90E	OQ911544
6	Hunan, Chenzhou	HNA	2020/09	24.97N/112.90E	OQ911548
7	Hunan, Chenzhou	HNB	2020/09	24.97N/112.90E	OQ911549
8	Hunan, Chenzhou	HNC	2020/09	24.97N/112.90E	OQ911550
9	Guangxi, Guilin	GXA	2020/08	25.90N/110.45E	OQ911545
10	Guangxi, Guilin	GXB	2020/08	25.90N/110.45E	OQ911546
11	Guangxi, Guilin	GXC	2020/08	25.90N/110.45E	OQ911547
12	Fujian, Wuyishan	FJA	2021/05	30.47N/103.13E	OQ911537
13	Fujian, Wuyishan	FJB	2021/05	30.47N/103.13E	OQ911538
14	Fujian, Wuyishan	FJB	2021/05	30.47N/103.13E	OQ911539
15	Guizhou, Zunyi	GZA	2011/11	28.28N/107.17E	OQ911551
16	Guizhou, Zunyi	GZB	2011/11	28.28N/107.17E	OQ911552
17	Sichuan, Ya’an	SCA	2021/06	30.20N/103.05E	OQ911533
18	Sichuan, Ya’an	SCB	2021/06	30.20N/103.05E	OQ911534
19	Sichuan, Ya’an	SCB	2021/06	30.20N/103.05E	OQ911535
20	Guangdong, Nanling	GDA	2021/05	24.90N/113.03E	OQ911530
21	Guangdong, Nanling	GDB	2021/05	24.90N/113.03E	OQ911531
22	Guangdong, Nanling	GDC	2021/05	24.90N/113.03E	OQ911532
23	Shaanxi, Yan’an	SXA	2019/07		OQ911536

Table S4. Genetic Distance (%), COI

	Genetic Distance (%)																						
**Name**	**SX-D**	**SX-E**	**SX-F**	**SC-C**	**SC-D**	**HN-B**	**HN-C**	**HN-D**	**GD-C**	**AH-B**	**AH-C**	**AH-D**	**ZJ-B**	**ZJ-C**	**ZJ-D**	**GX-D**	**GX-E**	**GX-F**	**GZ-D**	**GZ-E**	**GZ-F**	**FJ-D**	**FJ-E**
**SX-D**																							
**SX-E**	0.0																						
**SX-F**	0.2	0.2																					
**SC-C**	12.0	12.0	11.8																				
**SC-D**	11.8	11.8	11.6	0.2																			
**HN-B**	10.6	10.6	10.7	12.9	12.7																		
**HN-C**	10.5	10.5	10.7	12.7	12.5	2.2																	
**HN-D**	10.4	10.4	10.6	12.6	12.4	2.0	0.5																
**GD-C**	10.0	10.0	10.2	12.2	12.0	2.0	0.5	0.3															
**AH-B**	12.3	12.3	12.1	13.7	13.5	9.8	10.3	10.2	9.8														
**AH-C**	12.0	12.0	11.8	13.3	13.1	10.2	10.5	10.4	10.0	0.8													
**AH-D**	12.3	12.3	12.1	13.7	13.5	9.8	10.3	10.2	9.8	0.3	0.8												
**ZJ-B**	11.8	11.8	12.0	12.7	12.5	9.1	9.4	9.3	8.9	1.2	1.1	1.2											
**ZJ-C**	11.6	11.6	11.8	13.1	12.9	9.5	9.8	9.6	9.3	1.4	0.9	1.4	1.1										
**ZJ-D**	12.3	12.3	12.1	13.7	13.5	9.8	10.3	10.2	9.8	0.3	0.8	0.3	1.2	1.4									
**GX-D**	14.5	14.5	14.3	14.9	14.7	12.6	12.9	12.4	12.8	13.5	13.9	13.5	13.5	13.5	13.5								
**GX-E**	14.5	14.5	14.3	14.9	14.7	12.6	12.9	12.4	12.8	1.35	13.9	13.5	13.5	13.5	13.5	0.0							
**GX-F**	14.5	14.5	14.3	14.9	14.7	12.6	12.9	12.4	12.8	13.5	13.9	13.5	13.5	13.5	13.5	0.0	0.0						
**GZ-D**	11.6	11.6	11.8	14.6	14.4	12.0	12.3	11.8	11.8	12.6	12.6	12.2	11.5	12.0	12.6	13.7	13.7	13.7					
**GZ-E**	11.6	11.6	11.8	14.6	14.4	12.0	12.3	11.8	11.8	12.6	12.6	12.2	11.5	12.0	12.6	13.7	13.7	13.7	0.0				
**GZ-F**	11.6	11.6	11.8	14.6	14.4	12.0	12.3	11.8	11.8	12.6	12.6	12.2	11.5	12.0	12.6	13.7	13.7	13.7	0.0	0.0			
**FJ-D**	12.9	12.9	13.1	15.4	15.2	12.6	12.9	12.4	12.4	13.6	13.6	13.2	12.4	12.6	13.6	14.4	14.4	14.4	1.2	1.2	1.2		
**FJ-E**	11.4	11.4	11.6	14.4	14.2	12.2	12.5	12.0	12.0	13.0	13.0	12.6	11.8	12.4	13.0	14.0	14.0	14.0	1.5	1.5	1.5	2.8	

Table S5. Genetic Distance (%), 18S

	Genetic Distance (%)															
**Name**	**SX-D**	**SC-B**	**SC-C**	**SC-D**	**GD-B**	**GD-D**	**ZJ-B**	**ZJ-C**	**ZJ-D**	**GX-D**	**GX-E**	**GZ-D**	**GZ-E**	**GZ-F**	**FJ-D**	**FJ-E**
**SX-D**																
**SC-B**	3.52															
**SC-C**	3.52	0.00														
**SC-D**	3.52	0.00	0.00													
**GD-B**	3.03	2.22	2.22	2.22												
**GD-D**	3.52	2.22	2.22	2.22	0.95											
**ZJ-B**	7.05	7.74	7.74	7.74	6.88	6.54										
**ZJ-C**	6.54	7.22	7.22	7.22	6.37	6.03	0.79									
**ZJ-D**	6.54	7.22	7.22	7.22	6.37	6.03	0.79	0.00								
**GX-D**	7.58	8.80	8.80	8.80	8.10	7.75	8.28	8.11	8.11							
**GX-E**	7.23	8.45	8.45	8.45	7.75	7.41	8.28	7.76	7.76	1.26						
**GZ-D**	3.69	4.68	4.68	4.68	4.35	4.68	6.20	5.69	5.69	6.89	6.20					
**GZ-E**	2.71	4.02	4.02	4.02	3.69	4.02	5.86	5.35	5.35	6.71	6.37	0.94				
**GZ-F**	2.71	4.02	4.02	4.02	3.69	4.02	5.86	5.35	5.35	6.71	6.37	0.94	0.00			
**FJ-D**	2.71	4.02	4.02	4.02	3.69	4.02	5.86	5.35	5.35	7.06	6.72	1.58	0.63	0.63		
**FJ-E**	2.87	3.86	3.86	3.86	3.53	3.85	5.69	5.18	5.18	6.89	6.55	1.42	0.47	0.47	0.16	

Table S6. Genetic Distance (%), ITS2

	Genetic Distance (%)																					
**Name**	**SX-D**	**SC-B**	**SC-C**	**SC-D**	**HN-B**	**HN-C**	**HN-D**	**GD-B**	**GD-C**	**GD-D**	**AH-B**	**AH-C**	**AH-D**	**ZJ-B**	**ZJ-C**	**GX-D**	**GX-E**	**GX-F**	**GZ-D**	**GZ-F**	**FJ-D**	**FJ-E**	**FJ-F**
**SX-D**																							
**SC-B**	6.4																						

**SC-C**	6.7	0.3																				
**SC-D**	6.4	0.0	0.3																			
**HN-B**	8.6	7.6	8.0	7.6																		
**HN-C**	9.2	8.3	8.6	8.3	0.6																	
**HN-D**	8.9	8.0	8.3	8.0	0.3	0.3																
**GD-B**	5.7	5.1	5.4	5.1	7.6	8.3	8.0															
**GD-C**	5.7	5.4	5.7	5.4	5.1	5.1	4.8	3.0														
**GD-D**	4.8	4.8	5.1	4.8	7.3	7.9	7.6	1.5	2.7													
**AH-B**	9.3	9.2	9.6	9.2	8.3	8.6	8.6	9.9	7.6	9.3												
**AH-C**	7.6	7.6	7.9	7.6	6.4	6.7	6.7	8.3	6.0	7.6	1.8											
**AH-D**	7.6	7.6	7.9	7.6	6.4	6.7	6.7	8.3	6.0	7.6	1.8	0.0										
**ZJ-B**	7.0	5.7	6.0	5.7	4.5	5.1	4.8	6.4	4.2	5.7	4.2	2.4	2.4									
**ZJ-C**	7.0	6.4	6.7	6.4	5.1	5.7	5.4	7.0	4.8	6.4	4.2	2.4	2.4	0.6								
**GX-D**	10.2	7.3	7.6	7.3	6.7	7.3	7.0	9.6	7.0	8.9	9.3	7.3	7.3	4.8	5.4							
**GX-E**	10.6	7.6	8.0	7.6	7.0	7.6	7.3	10.0	7.3	9.3	9.6	7.6	7.6	5.1	5.7	0.3						
**GX-F**	10.6	7.6	8.0	7.6	7.0	7.6	7.3	10.0	7.3	9.3	9.6	7.6	7.6	5.1	5.7	0.3	0.6					
**GZ-D**	9.6	5.7	6.0	5.7	9.2	9.9	9.6	8.0	7.9	7.0	10.9	10.2	10.2	8.3	8.9	9.6	9.9	9.9				
**GZ-F**	8.9	5.1	5.4	5.1	8.3	8.9	8.6	7.3	7.3	6.4	11.9	9.9	9.9	7.9	8.6	9.3	8.9	9.6	2.1			
**FJ-D**	5.4	3.3	3.6	3.3	6.7	7.3	7.0	4.5	4.5	3.6	8.9	7.3	7.3	5.4	6.0	7.0	7.3	7.3	6.0	5.4		
**FJ-E**	5.4	3.3	3.6	3.3	6.4	7.0	6.7	4.5	4.5	3.6	8.3	7.0	7.0	5.1	5.7	6.7	7.0	7.0	5.4	5.1	0.6	
**FJ-F**	5.4	3.0	3.3	3.0	5.7	6.4	6.0	4.5	4.5	3.6	8.3	6.4	6.4	4.5	5.1	6.0	6.4	6.4	5.4	4.5	0.9	0.6

Table S7. Analysis of molecular variance (AMOVA) of mitochondrial DNA COI

for 23 individuals in 9 *R. sinensis* populations

Source of variation	df	Sum of squares	Variance components	% of variation	Fst
Among populations	8	671.529	32.23692	93.35	0.93347
Within populations	14	32.167	2.29762	6.65	
Total	22	703.696	34.53454		

Table S8. Analysis of molecular variance (AMOVA) of 18S rDNA

for 17 individuals in 8 *R. sinensis* populations

Source of variation	df	Sum of squares	Variance components	% of variation	Fst
Among populations	7	292.814	19.28106	92.13	0.92125
Within populations	9	14.833	1.64815	7.87	
Total	16	307.647	20.92921		

Table S9. Analysis of molecular variance (AMOVA) of ITS2 for 23 individuals in 9 *R. sinensis* populations

Source of variation	df	Sum of squares	Variance components	% of variation	Fst
Among populations	8	208.659	9.67346	85.93	0.85934
Within populations	14	22.167	1.58333	14.07	
Total	22	236.826	11.25680		

Table S10. The F_{st} value and genetic flow based on COI gene among 9 geo-populations of *R. sinensis*

Population	Guangdong	Sichuan	Shaanxi	Fujian	Anhui	Zhejiang	Guangxi	Hunan	Guizhou
Guangdong		0.00699	0.00549	0.16364	0.03550	0.08108	0.00000	inf	0.00000
Sichuan	0.98621		0.00553	0.06129	0.01948	0.03905	0.00194	0.05072	0.00196
Shaanxi	0.98913	0.98907		0.04888	0.01651	0.03213	0.00198	0.04545	0.00238
Fujian	0.75342	0.89080	0.91095		0.06336	0.08955	0.03896	0.10345	1.50000
Anhui	0.93370	0.96250	0.96804	0.88753		13.50000	0.01271	0.06351	0.01382
Zhejiang	0.86047	0.92756	0.93963	0.84810	0.03571		0.02632	0.08960	0.02941
Guangxi	1.00000	0.99614	0.99606	0.92771	0.97521	0.95000		0.03555	0.00000
Hunan	-0.66667	0.90789	0.91667	0.82857	0.88730	0.84803	0.93363		0.03713
Guizhou	1.00000	0.99610	0.99526	0.25000	0.97309	0.94444	1.0000	0.93088	

Table S11. The F_{st}-*P* value and Exact test results based on COI gene among 9 geo-populations of *R. sinensis*

Population	Guangdong	Sichuan	Shaanxi	Fujian	Anhui	Zhejiang	Guangxi	Hunan	Guizhou
Guangdong		1.00000	0.50321	1.00000	1.00000	1.00000	0.24867	1.00000	0.24932
Sichuan	0.360±0.04		0.39832	1.00000	1.00000	1.00000	0.09965	1.00000	0.09903
Shaanxi	0.279±0.05	0.117±0.03		0.40435	0.40548	0.40444	0.09795	0.39935	0.10118
Fujian	0.306±0.04	0.315±0.05	0.108±0.03		1.00000	1.00000	0.10045	1.00000	0.10124
Anhui	0.306±0.05	0.063±0.02	0.06±0.02	0.153±0.03		1.00000	0.10116	1.00000	0.09912
Zhejiang	0.189±0.04	0.153±0.02	0.108±0.04	0.144±0.04	0.378±0.04		0.09805	1.00000	0.10285
Guangxi	0.189±0.04	0.081±0.03	0.081±0.03	0.07±0.02	0.135±0.04	0.081±0.04		0.10155	0.10281
Hunan	0.991±0.00	0.081±0.03	0.108±0.02	0.153±0.03	0.072±0.02	0.135±0.03	0.072±0.04		0.10131
Guizhou	0.324±0.02	0.072±0.02	0.144±0.03	0.108±0.02	0.036±0.02	0.126±0.03	0.090±0.03	0.063±0.02	

Table S12. The F_{st} value and genetic flow based on ITS2 gene among 9 geo-populations of *R. sinensis*

Population	Guangdong	Sichuan	Shaanxi	Fujian	Anhui	Zhejiang	Guangxi	Hunan	Guizhou
Guangdong		0.16667	0.40000	0.29245	0.15245	0.22500	0.09859	0.13043	0.23390
Sichuan	0.75000		0.01613	0.07895	0.04730	0.02901	0.02083	0.01974	0.09135
Shaanxi	0.55556	0.96875		0.07447	0.08824	0.04762	0.02062	0.02410	0.15217
Fujian	0.63095	0.86364	0.87037		0.07480	0.07186	0.04472	0.04622	0.14384
Anhui	0.76623	0.91358	0.85000	0.86986		0.24590	0.05556	0.06349	0.08721
Zhejiang	0.68966	0.94516	0.91304	0.87435	0.67033		0.04839	0.05048	0.09783
Guangxi	0.83529	0.96000	0.96040	0.91791	0.90000	0.91176		0.02985	0.05902
Hunan	0.79310	0.96203	0.95402	0.91538	0.88732	0.90830	0.94366		0.06205
Guizhou	0.68129	0.84552	0.76667	0.77660	0.85149	0.83636	0.89442	0.88959	

Table S13. The F_{ST}-P value and Exact test results based on ITS2 gene among 9 geo-populations of *R. sinensis*

Population	Guangdong	Sichuan	Shaanxi	Fujian	Anhui	Zhejiang	Guangxi	Hunan	Guizhou
Guangdong		0.40101	1.00000	1.00000	0.39661	1.00000	1.00000	1.00000	1.00000
Sichuan	0.045±0.02		0.50620	0.39304	0.20065	0.39757	0.40504	0.39813	0.39957
Shaanxi	0.991±0.00	0.991±0.00		1.00000	0.49363	1.00000	1.00000	1.00000	1.00000
Fujian	0.081±0.03	0.117±0.03	0.234±0.03		0.40413	1.00000	1.00000	1.00000	1.00000
Anhui	0.090±0.02	0.144±0.02	0.351±0.05	0.081±0.03		0.40144	0.40057	0.39876	0.40249
Zhejiang	0.162±0.04	0.099±0.03	0.315±0.06	0.117±0.03	0.081±0.02		1.00000	1.00000	1.00000
Guangxi	0.081±0.02	0.090±0.03	0.261±0.05	0.063±0.02	0.063±0.02	0.063±0.02		1.00000	1.00000
Hunan	0.072±0.02	0.081±0.03	0.189±0.03	0.063±0.02	0.099±0.02	0.126±0.04	0.207±0.05		1.00000
Guizhou	0.126±0.02	0.126±0.03	0.369±0.06	0.108±0.03	0.180±0.02	0.387±0.04	0.117±0.04	0.036±0.02	

Table S14. The F_{st} value and genetic flow based on 18S gene among 7 geo-populations of *R. sinensis*

Population	Guangdong	Sichuan	Shaanxi	Fujian	Zhejiang	Guangxi	Guizhou
Guangdong		0.08571	0.20690	0.08750	0.06096	0.08537	0.11351
Sichuan	0.85366		0.00000	0.00691	0.01923	0.02673	0.04110
Shaanxi	0.70732	1.00000		0.03030	0.04425	0.10526	0.13333
Fujian	0.85106	0.98636	0.94286		0.04112	0.05921	0.56250
Zhejiang	0.89133	0.96296	0.91870	0.92401		0.05749	0.05914
Guangxi	0.85417	0.94926	0.82609	0.89412	0.89688		0.07524
Guizhou	0.81498	0.92405	0.78947	0.47059	0.89423	0.86921	

Table S15. The F_{st}-P value and Exact test results based on 18S gene among 7 geo-populations of *R. sinensis*

Population	Guangdong	Sichuan	Shaanxi	Fujian	Zhejiang	Guangxi	Guizhou
Guangdong		0.09900	1.00000	1.00000	0.39569	1.00000	0.39976
Sichuan	0.072±0.02		0.24878	0.10370	0.10190	0.09899	0.09769
Shaanxi	0.991±0.00	0.991±0.00		1.00000	0.50229	1.00000	0.49453
Fujian	0.324±0.04	0.126±0.02	0.315±0.05		0.39828	1.00000	0.39648
Zhejiang	0.108±0.03	0.054±0.02	0.225±0.04	0.162±0.02		0.39825	0.19929
Guangxi	0.423±0.05	0.153±0.03	0.306±0.03	0.342±0.04	0.117±0.03		0.40431
Guizhou	0.108±0.03	0.081±0.03	0.288±0.05	0.153±0.03	0.099±0.02	0.054±0.02	

Table S16. Haplotype distribution (frequencies) through *R. sinensis* populations

Number	Collection Locations	code	Sample size	Haplotypes (no. individuals)
1	Anhui	AH	3	Hap-8(1), Hap-9(1), Hap-10(1)
2	Zhejiang	ZJ	3	Hap-11(1), Hap-12(1), Hap-13(1)
3	Hunan	HN	3	Hap-15(1), Hap-16(1), Hap-17(1)
4	Guangxi	GX	3	Hap-14(3)
5	Fujian	FJ	2	Hap-6(1), Hap-7(1)
6	Guizhou	GZ	3	Hap-18(3)
7	Sichuan	SC	2	Hap-2(1), Hap-3(1)
8	Guangdong	GD	1	Hap-1(1)
9	Shaanxi	SX	3	Hap-4(2), Hap-5(1)

Table S17. Haplotype distribution (frequencies) through *R. sinensis* populations

Number	Collection Locations	code	Sample size	Haplotypes (no.individuals)
1	Anhui	AH	1	Hap-7(1)
2	Zhejiang	ZJ	3	Hap-8(1), Hap-9(2)
3	Guangxi	GX	2	Hap-10(1), Hap-11(1)
4	Fujian	FJ	2	Hap-5(1), Hap-6(1)
5	Guizhou	GZ	3	Hap-12(1), Hap-13(2)
6	Sichuan	SC	3	Hap-3(3)
7	Guangdong	GD	2	Hap-1(1), Hap-2(1)
8	Shaanxi	SX	1	Hap-4(1)

Table S18. Haplotype distribution (frequencies) through *R. sinensis* populations

Number	Collection Locations	code	Sample size	Haplotypes (no.individuals)
1	Anhui	AH	3	Hap-10(1), Hap-11(2)
2	Zhejiang	ZJ	2	Hap-12(1), Hap-13(1)
3	Hunan	HN	3	Hap-17(1), Hap-18(1), Hap-19(1)
4	Guangxi	GX	3	Hap-14(1), Hap-15(1)Hap-16(1)
5	Fujian	FJ	3	Hap-7(1), Hap-8(1), Hap-9(1)
6	Guizhou	GZ	2	Hap-20(1), Hap-21(1)
7	Sichuan	SC	3	Hap-4(2), Hap-5(1)
8	Guangdong	GD	3	Hap-1(1), Hap-2(1), Hap-3(1)
9	Shaanxi	SX	1	Hap-6(1)
